# Methanotroph populations and CH_4_ oxidation potentials in high-Arctic peat are altered by herbivory induced vegetation change

**DOI:** 10.1093/femsec/fiaa140

**Published:** 2020-07-08

**Authors:** Edda M Rainer, Christophe V W Seppey, Alexander T Tveit, Mette M Svenning

**Affiliations:** Department of Arctic and Marine Biology, UiT – The Arctic University of Norway, Tromsø, Norway; Department of Arctic and Marine Biology, UiT – The Arctic University of Norway, Tromsø, Norway; Department of Arctic and Marine Biology, UiT – The Arctic University of Norway, Tromsø, Norway; Department of Arctic and Marine Biology, UiT – The Arctic University of Norway, Tromsø, Norway

**Keywords:** methane oxidation, *Methylobacter*, high-Arctic peatland soils, grazing pressure, active MOB community

## Abstract

Methane oxidizing bacteria (methanotrophs) within the genus *Methylobacter* constitute the biological filter for methane (CH_4_) in many Arctic soils. Multiple *Methylobacter* strains have been identified in these environments but we seldom know the ecological significance of the different strains. High-Arctic peatlands in Svalbard are heavily influenced by herbivory, leading to reduced vascular plant and root biomass. Here, we have measured potential CH_4_ oxidation rates and identified the active methantrophs in grazed peat and peat protected from grazing by fencing (exclosures) for 18 years. Grazed peat sustained a higher water table, higher CH_4_ concentrations and lower oxygen (O_2_) concentrations than exclosed peat. Correspondingly, the highest CH_4_ oxidation potentials were closer to the O_2_ rich surface in the grazed than in the protected peat. A comparison of 16S rRNA genes showed that the majority of methanotrophs in both sites belong to the genus *Methylobacter*. Further analyses of *pmoA* transcripts revealed that several *Methylobacter* OTUs were active in the peat but that different OTUs dominated the grazed peat than the exclosed peat. We conclude that grazing influences soil conditions, the active CH_4_ filter and that different *Methylobacter* populations are responsible for CH_4_ oxidation depending on the environmental conditions.

## Teaser

Grazing in high-Arctic peatlands leads to soil ecosystem changes selecting for niche-adapted methanotrophs.

## INTRODUCTION

High-Arctic peatlands store large amounts of organic carbon that is a source for microbial production of the greenhouse gas methane (CH_4_). As a result of climate change, these peatlands are exposed to increased temperatures, changes in precipitation, herbivory and vegetation composition that might lead to increased CH_4_ production rates (Parish *et al*. 2008; Sjögersten *et al*. 2011). Methane oxidizing bacteria (MOB), or methanotrophs, act as the dominant biological CH_4_ filter in peat soils, consuming CH_4_ produced in deeper anaerobic peat before it is released to the atmosphere (Reay, Smith and Hewitt 2007). MOB are a diverse group of bacteria, found within the classes *Gammaproteobacteria, Alphaproteobacteria* and *Verrucomicrobia* (Hanson and Hanson 1996; Knief 2015). The abundances and distribution of most MOB can be assessed by quantification and analysis of the *pmoA* gene which encodes the β**-**subunit of the particulate CH_4_ monooxygenase (McDonald *et al*. 2008; Knief 2015). A broad diversity of MOB has been identified using *pmoA*, making it possible to evaluate the habitat preferences of different MOB (Knief 2015). Many cold ecosystems with a neutral pH are found to be dominated by MOB within *Gammaproteobacteria* (Wartiainen, Hestnes and Svenning 2003; Börjesson, Sundh and Svensson 2004; Wartiainen *et al*. 2006; Martineau, Whyte and Greer 2010; Graef *et al*. 2011). Among these, MOB within the genus *Methylobacter* are identified as the main CH_4_ oxidizers in many freshwater wetlands (Yun *et al*. 2010; Tveit *et al*. 2013; Singleton *et al*. 2018; Smith *et al*. 2018; Zhang *et al*. 2019). Members of this group and other gammaproteobacterial MOB were also found in association with *Sphagnum sp*. mosses and vascular plants in a temperate peatland with their composition and activities being directly related to the plant biodiversity (Stępniewska *et al*. 2018). Methanotroph communities and their activities are known to be influenced by CH_4_ concentration, pH, O_2_ concentration, temperature, nitrogen concentration and copper availability (Amaral and Knowles 1995; Semrau, DiSpirito and Yoon 2010; Ho *et al*. 2013). It has been suggested that O_2_ distribution plays a crucial role and may explain niche-adaptation in freshwater lakes (Biderre-Petit *et al*. 2011; Blees *et al*. 2014; Oshkin *et al*. 2015; Mayr *et al*. 2020) and flooded paddy soils (Reim *et al*. 2012). However, the ecology of large OTU numbers within e.g. *Methylobacter* and other gammaproteobacterial MOB (Tsutsumi *et al*. 2011; He *et al*. 2012; Beck *et al*. 2013; Oshkin *et al*. 2014; Knief 2015; Oswald *et al*. 2015; Bornemann *et al*. 2016), is mostly unknown. As a result, it is still difficult to explain the co-existence of many closely related OTUs within a defined ecosystem.

Grazing by geese and reindeer reduces the biomass of grasses and herbs. Warmer winters in temperate regions, increased food availability due to changes in agriculture and protection from hunting has led to an increase in the total geese population (Hessen *et al*. 2017). In Solvatn (Ny Ålesund, Svalbard, Northern Norway) experimental peat plots protected from geese herbivory by fences doubled the vegetation over the course of nine years, leading to increased peatland carbon uptake (Sjögersten *et al*. 2011). During peat formation, the plant cover and its roots influence the physical properties of the soil such as porosity and pore direction (Kruse, Lennartz and Leinweber 2008). In addition, root exudates stimulate microbial communities within the soil and the exudates from different plant types (Bardgett *et al*. 2013), potentially create niches for different microorganisms. Thus, herbivory or its absence, may have a substantial effect on the soil structure, biology and chemistry.

Solvatn and adjacent peatlands close to Ny-Ålesund have been studied thoroughly during the last 15 years with emphasis on the CH_4_ cycle, showing that substantial potentials for CH_4_ production and oxidation exist in these soils (Høj, Olsen and Torsvik 2005; Graef *et al*. 2011; Tveit *et al*. 2013, 2015). However, the effect of changes in herbivory on the CH_4_ cycles were not studied. Here, in a comparison of 18-year old exclosures (Sjögersten *et al*. 2011) and nearby grazed sites, we have addressed how the microbial CH_4_ filter is affected by intensive herbivore grazing over years. Specifically, we investigated the relationship between altered soil properties, potential CH_4_ oxidation rates and the active methanotroph communities by 16S rRNA gene and *pmoA* transcript amplicon analyses.

## MATERIALS AND METHODS

### Field site and sampling

**Figure 1.**
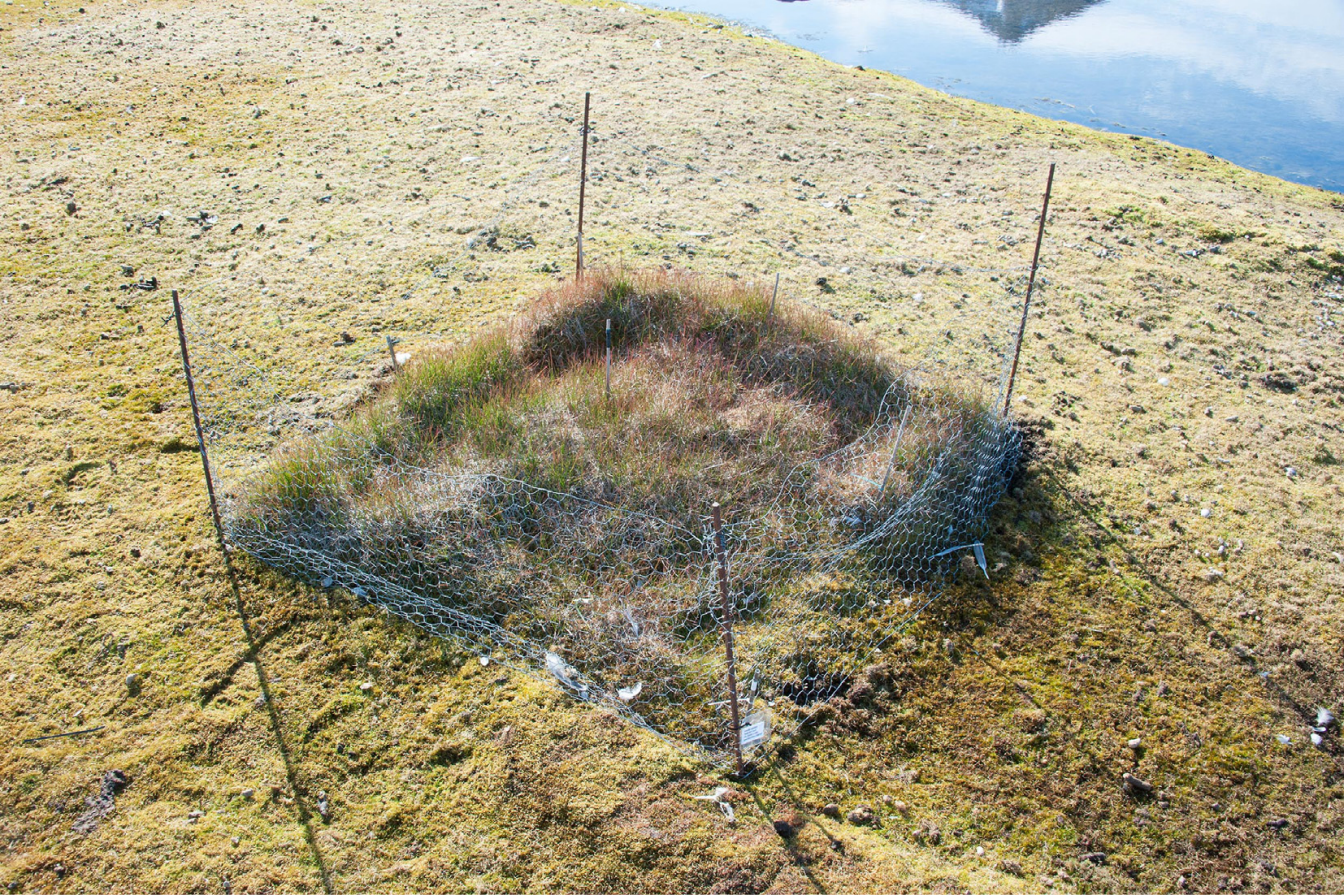
Exclosure at site SV1, Solvatn peatland, Ny Ålesund, Svalbard. Exclosure size is 1 × 1 m. It has been protected from grazing by a wire fence for 18 years.

The Solvatn peatland (N78°55.550, E11°56.611) is located close to Ny Ålesund, Svalbard. It is heavily grazed by Barnacle geese (*Branta leucopsis*) and is dominated by brown mosses, primarily *Calliergon richardsonii* (Solheim, Endal and Vigstad 1996). Exclosures established in 1998 protect parts of the peatland vegetation from geese grazing (Sjögersten *et al*. 2011) allowing growth of vascular plants that are otherwise suppressed by grazing (Fig. 1). Two sampling sites from the Solvatn peatland were selected (SV1 and SV2), both of which were used by Sjögersten *et al*. (2011). Each site includes an exclosed plot and an adjacent grazed plot. Two field campaigns were conducted in summer, during the active growing season (August 2015 and 2016), while one field campaign was conducted immediately after snowmelt (June 2016). Below, we refer to these time points as summer 2015, spring 2016 and summer 2016.

In each plot, two blocks (approx. 30 × 30 × 20 cm) were cut from the peat soil and kept cool throughout transportation to the on-site laboratory, approximately 600 m away from the field site. Each block was separated in three vertical sections designated A, B and C (approx. 30 × 10 × 20 cm).

Section A was frozen at −20 °C and transported to the laboratory at UiT, The Arctic University of Norway, where it was used for the determination of water content and soil organic matter (SOM).

Section B and C were then divided in seven horizontal layers (0.5–8 cm for the upper six layers, 8–12 cm for the lowest layer), which served as material for the further analyses. The top layers (soil surface) of all plots were composed only of plants, whereas the layers two to seven were composed of partly decomposed peat. In the exclosed plots, the top 2–3 cm below the vegetation was a mixture of roots and peat soil. In the grazed plots, few or no living roots were observed.

Section B layers were used to measure CH_4_ oxidation potentials *ex situ* (see next section). At the end of these measurements, peat samples from each microcosm were collected in 15 ml plastic tubes (VWR High-Performance polypropylene centrifuge tubes), flash frozen in liquid nitrogen (N_2_) and stored at −80 °C.

Section C layers were transferred to sterile 15 ml plastic tubes (VWR High-Performance polypropylene centrifuge tubes), flash frozen in liquid N_2_ immediately after arrival on the on-site laboratory and stored at −80 °C.

All soil samples from section B and C were shipped to the laboratory at UiT, the Arctic University of Norway, and stored at −80 °C until further processing. An overview of the sampling design and the respective analyses for each sampling is provided in Table S1 (Supporting Information).

### Environmental characterization

Prior to soil water content and SOM analysis, section A from each sampling campaign was thawed in a cold room (8 °C) and separated into horizontal layers as described for sections B and C. Soil water content was determined gravimetrically by drying 10 g of peat at 150 °C over night. The dried peat soil was then incinerated at 450 °C and the amount of burnt peat matter was determined gravimetrically to deduce the amount of SOM.

O_2_ concentration and temperature were measured *in situ*, using an optical O_2_ sensor and thermometer (Fibox 4 Optode, Presens, Germany). Measurements were taken at the soil surface and at 5 cm intervals down to 20 cm depth.

To measure *in situ* CH_4_ concentrations, pore water samples were extracted at 5, 10, 15 and 20 cm depth below the vegetation as described in Liebner *et al*. (2012). Briefly, we extracted 5 ml pore water using perforated brass rods and injected the pore water into 20 ml serum vials, which had been added 0.1 ml 1 M HCl and flushed with N_2_. Headspace CH_4_ concentrations in these vials were measured using a GC-FID (SRI Instruments, CA, USA). Gas samples were retrieved using a pressure tight syringe (Vici Precision Sampling, LA, USA) and injected directly onto a GC-FID with a HayeSep D packed column (SRI Instruments, CA, USA). The instrument sensitivity was set to its maximum and the elution time for CH_4_ was 1.8 min.

From the headspace concentrations we could estimate the mass of headspace CH_4_ by the ideal gas law. Further, when accounting for the dissolved CH_4_ at room temperature (21 °C) and serum vial pressure using Henrys law constant for solubility of CH_4_ in water, the headspace CH_4_ content inside the serum vials equals the pore water CH_4_ content.

### Microcosm experiment for potential CH_4_ oxidation *ex situ* along soil gradients

To measure potential CH_4_ oxidation, approximately 15 g of each peat soil layer from section B were transferred to sterile 175 ml serum bottles and closed using Butyl rubber stoppers (Wheaton, Niemann *et al*. 2015) and aluminium crimp caps. CH_4_ was added to obtain headspace concentrations of 0.5%–0.6% CH_4_ (injection of 1 ml CH_4_, 100 v/v %). Such high concentrations were chosen to ensure CH_4_ availability for the MOB during the incubation. We acknowledge a potential selective pressure towards low affinity MOB but we considered this bias as preferable to several perturbations caused by a large number of CH_4_ injections or periods of CH_4_ starvation. A volume of 34 ml air was added to obtain overpressure in the microcosms to enable easier sampling. Gas measurements were conducted from the headspace of the incubation bottles immediately after CH_4_ injection followed by four subsequent time-points at regular intervals during a maximum of 45 h of incubation at 8 °C using a GC-FID (SRI Instruments, CA, USA). Details for the headspace sampling and the GC program are described in the section ‘Environmental characterization’ above. From the headspace CH_4_ concentrations measured at each time point we calculated the CH_4_ oxidation rate.

### Nucleic acid extraction/16S rRNA gene and *pmoA* amplicon sequencing

Nucleic acids were extracted from the *in situ* peat soil layers (section C, spring and summer 2016). For extraction, we selected the layers with maximum CH_4_ oxidation activity (0–2 cm depth in grazed plots, 4–8 cm depth in exclosed plots). From these extracts, we purified both DNA and RNA to identify the *in situ* bacterial community and active MOB community by 16S rRNA gene and *pmoA* transcript sequencing, respectively.

Additionally, nucleic acids were extracted from the samples collected and frozen at the end of the 45-hours incubation period (section B from summer 2015). In correspondence with the *in situ* peat soil samples, we selected layers with maximal CH_4_ oxidation activity. From these extracts, RNA was purified and used for sequencing of *pmoA* transcripts. This was done to identify the active MOB community responsible for CH_4_ oxidation during the microcosm experiment.

All samples were ground with mortar and pestle in liquid N_2_. Total nucleic acids were purified in duplicates from 0.2 g of each ground peat soil sample using a phenol/chloroform extraction protocol (Urich *et al*. 2008; Tveit *et al*. 2013). The duplicates of nucleic acids were mixed and then split in two samples, one for DNA purification and one for RNA purification.

DNA was purified by removing RNA with RNase A/T1 (Thermo Fisher Scientific, Waltham, MA/USA), followed by phenol/chloroform extraction and ethanol precipitation. Quality of DNA was assessed by Nanodrop and gel electrophoresis. DNA amplification was confirmed for the 16S rRNA gene using the 27F/1492R primer pair (Lane 1991). For 16S rRNA gene sequencing the V3-V4 region was targeted (Klindworth *et al*. 2013) using the Illumina MiSeq platform at IMGM Laboratories, Germany. The 16S rRNA gene amplicons were generated by a 2-step target-specific (TS)-PCR using 1 ng DNA as template for 25 cycles followed by an 8-cycle index PCR using 1 µL TS PCR product as template. The Q5® High Fidelity polymerase from NEB (Ipswich, MA, USA) was used for both PCRs and a negative control as well as a Mock community were amplified and sequenced in parallel to ensure sufficient quality.

To purify RNA, DNA was removed (RQ1 DNase, Promega), followed by RNA clean-up (MegaClear, Ambion) and ethanol precipitation. The RNA quality was assessed by Nanodrop and gel electrophoresis. RNA was reverse-transcribed (Superscript IV, Thermo Fisher) and the cDNA template was verified for the *pmoA* gene using the A189F/mb661R primer pair (Costello and Lidstrom 1999). The cDNA samples were sequenced using Illumina MiSeq and the two *pmoA* targeting primer pairs, A189F/mb661R and A189F/A682R (Holmes *et al*. 1995; Costello and Lidstrom 1999) at IMGM Laboratories, Germany. The *pmoA* gene amplicons were generated by a 2-step TS-PCR using 10 ng cDNA as template for 25 cycles followed by a 12-cycle index PCR using 1 µL from the TS PCR products. The polymerase used was the Q5® High-Fidelity polymerase from NEB (Ipswich, MA, USA). Both a negative control and a Mock Community were amplified and sequenced in parallel to the samples to ensure sufficient quality.

### Bioinformatics

#### Databases

Taxonomic assignment of OTUs for each of the three sequenced communities was done with a de-replicated database with sequences trimmed according to the primers used in this study. The sequences used for the *pmoA* database were retrieved from a published collection of *pmoA* sequences (Wen, Yang and Liebner 2016) and complemented with three Arctic *Methylobacter* sequences originating from Svalbard (GenBank id = AJ414658.1, KC878619.1, G7 Arctic mine isolate (genome not published)). The V3-V4 16S rRNA gene database was built from fragments of the SILVA 128 SSU database (Quast *et al*. 2013) (downloaded the 1st of October 2017). Both databases were trimmed according to the corresponding primers and de-replicated with a custom Perl script (https://github.com/cseppey/bin_src_my_prog/tree/master/perl/sel_db.pl).

#### Sequence data analyses

For each of the environmental sequence datasets, reads were merged using the program Flash (v. 1.2.8; (Magoč and Salzberg 2011)). Good quality sequences were filtered using a custom script (https://github.com/cseppey/bin_src_my_prog/tree/master/cpp/qualCheck.cpp) by keeping only sequences without any window of 50 nucleotides with an average phred score below 20 prior to trimming the primers (https://github.com/cseppey/bin_src_my_prog/tree/master/perl/trim_primer.pl). Chimeras were removed using the program Vsearch (v. 2.4.4; (Rognes *et al*. 2016)) comparing the environmental sequences between them (de novo approach), as well as by comparing the sequences against the corresponding database (for *pmoA* primers: (Wen, Yang and Liebner 2016); for V3-V4 primer: SILVA 128). After trimming the primers the *pmoA* sequences were expected to start with the nucleotides 188–190 (TCG: serine) and finish with the nucleotides 658–660 (TAT: tyrosine) for the reversed primer mb661 or nucleotides 679–681 (TCG: serine) for the reversed primer A682R (Semrau *et al*. 1995). To avoid sequences containing frameshift mutations, thus incorrect open reading frames, sequences with a number of nucleotides not divisible by three and sequences containing a stop codon were removed.

OTUs were clustered from the processed environmental sequences using the program Swarm (v. 2.1.13; (Mahé *et al*. 2014)), and taxonomically assigned by using the best alignment between the dominant sequence of each OTU and the database using the program Ggsearch36 (v. 36.3.8f; (Pearson 2000)). The OTUs were finally selected according to their length (mb661: [465–474 basepairs (bp)], A682: [492–495 bp], V3-V4: [370–435 bp]) in order to remove obvious sequencing errors as well as to their taxonomic affiliation by discarding OTUs assigned to Archaea, chloroplast or mitochondria.

#### Statistical analyses

To reduce the noise caused by low relative abundances, we consider an OTU as absent of a sample if its relative abundance was < 0.001 in that sample. Prior to analyses, the three relative abundance community matrices were log normalized as previously described in (Anderson, Ellingsen and McArdle 2006) (function decostand, package vegan v. 2.5–2; (Oksanen *et al*. 2018)). The effect of factor (treatment i.e. grazing and sampling date), interaction between the factors and CH_4_ rate, while removing the effect of sites, were assessed through redundancy analysis (RDA) (function capscale, package vegan v.2.5–2; (Oksanen et al 2018)). The significance of the factors, factors interaction and CH_4_ rate, as well as the significance of the RDA axes were tested by a permutation test (10 000 permutations, function anova.cca, package vegan v. 2.5–2; (Oksanen *et al*. 2018)). To disentangle the effect of the interaction between grazing and sampling date, two other RDAs were calculated for each treatment. For each new RDA, the effects of sampling date, CH_4_ oxidation rate as well as the RDA axes were tested as for the RDA performed on the two treatments together.

The most representative OTUs of each treatment (bioindicators) were assessed using an indicator species analysis (indval; function indval, package labdsv v. 1.8–0; (Roberts 2016)) on the relative abundance community matrices. For each OTU in each treatment, a score is calculated, which is maximized if (i) the OTU is mostly found in the given treatment (high specificity) and (ii) is found in all samples of the given treatment (high fidelity). An OTU was selected as a bioindicator if the probability of a higher indicator value was < 0.001 on 10 000 permutations. All statistical analyses were performed in R (R Core Team 2018) and an overview of the sequence/OTU number at each step of the pipeline is found in the Table S2 (Supporting Information).

A phylogenetic tree was built from the bioindicator OTU sequences as well as closely related sequences retrieved from NCBI GenBank in order to better assess their taxonomy. The closely related sequences were retrieved by aligning (BLASTn) the bioindicator sequences against the NCBI nucleotide database and choosing the two highest scoring matches. In addition, a set of cultivated gammaproteobacterial MOB sequences was retrieved in addition to a set of *pmoA* sequences belonging to upland-soil cluster (USC)-gamma that served as an outgroup. Sequences were aligned in MEGA7 (Kumar, Stecher and Tamura 2016) using MUSCLE, choosing the UPGMB clustering (Edgar 2004). The length of the alignment was inspected visually for an overlap for all sequences and a section of 440 bp was chosen for phylogenetic analysis. A phylogenetic tree was constructed in MEGA7 using the neighbor-joining method with the Jukes-Cantor correction and 500 bootstraps (Kumar, Stecher and Tamura 2016). The tree was visualized using FigTree v1.4.4 (Rambaut 2018).

## RESULTS

### Soil parameters

Soil temperatures decreased with depth in both grazed and exclosed plots, and higher temperatures were measured in the summer seasons than in the spring season (Table S3, Supporting Information). At the soil surface, temperatures up to 16 °C were observed but temperatures varied substantially depending on air temperature and cloud cover (Fig. S1, Supporting Information). Below the surface, temperatures rarely exceeded 8 °C throughout the peat profile. Slightly warmer temperatures were recorded in grazed plots in the top 10 cm of the peat soil.

The decrease in O_2_ concentration with depth was similar in grazed (-0.51 ± 0.18 mg/L per cm depth) and exclosed plots (-0.35 ± 0.25 mg/L per cm depth). However, O_2_ concentrations in grazed plots dropped from 9.8–11.5 mg/L O_2_ at the surface to 1.7–9.5 mg/L O_2_ at 5 cm depth. No drop was observed in exclosed plots when comparing surface concentrations (10.4–11.6 mg/L O_2_) to 5 cm depth (9.6–12.1 mg/L O_2_) and a more gradual decrease in O_2_ concentration was observed (Fig. S2, Supporting Information).

**Figure 2.**
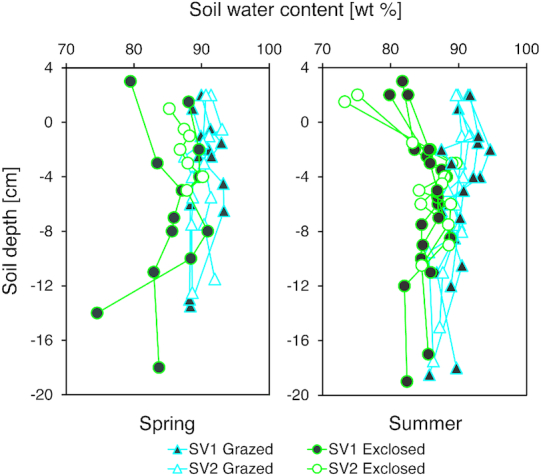
Soil water content in spring (left) and summer 2015 and 2016 (right), comparing grazed treatment (blue) and exclosed treatment (green). The water content was measured for the vegetation (>0 cm soil depth), at the soil/vegetation interface (0 cm) and until a soil depth of 20 cm (y-axis). Each point represents one measurement. Soil water content (x-axis) varied from 73 to 95 wt%. Soil depths were chosen in order to include visually distinguishable layers as well as many depths within the layers suspected to account for the majority of CH_4_ oxidation (0–10 cm soil depth).

Comparing the vegetation and the top 2 cm of peat soil, the water content was lower in exclosed plots (73.4–89.6 wt% H_2_O) than in grazed plots (88.7–94.7 wt% H_2_O) (Fig. 2), whereas between 2 and 10 cm below vegetation the water content was more similar for both environments. Overall, the soil water content measured in exclosed plots was 2%–15% lower than in grazed plots. SOM was slightly higher in exclosed peat (11.5 ± 3.2%) compared to grazed peat (8.3% ± 1.5%).

The *in situ* pore water CH_4_ concentrations were higher at 10 cm depth than 5 cm depth in the grazed plots. Similarly, the CH_4_ concentrations were higher at 20 cm depth than 15 cm depth in exclosed plots. Moreover, *in situ* CH_4_ pore water concentrations were consistently higher in grazed plots than exclosed plots (Fig. S3, Supporting Information).

### Potential CH_4_ oxidation

**Figure 3.**
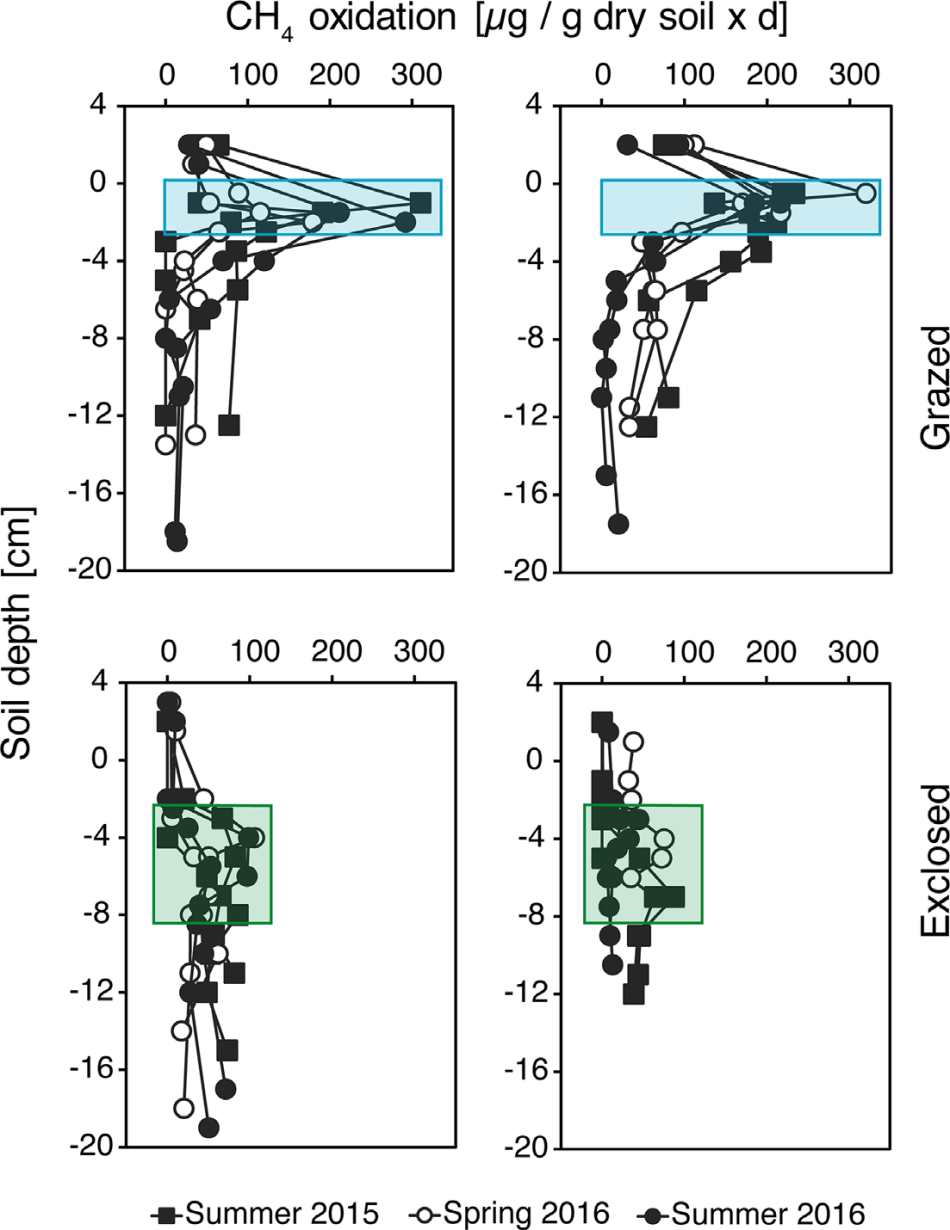
Potential CH_4_ oxidation (µg CH_4_ oxidized per g dry soil and per day) along vertical soils gradients for the grazed (two top figures) and exclosed treatments (two bottom figures). Filled symbols represent summer and open symbols represent spring. Each point represents one measurement. Oxidation rates from site SV1 and SV2 are shown on the left; and right- hand side respectively. The shaded area highlights the zone of maximum CH_4_ oxidation activity. CH_4_ oxidation measured above ground (i.e. vegetation) are > 0 on the y-axis, whereas below ground activity are < 0 on the y-axis.

Microcosm experiments were conducted *ex situ* to estimate the potential soil CH_4_ oxidation rates at different depths in grazed and exclosed plots, for different seasons (spring and summer) and years. The highest CH_4_ oxidation rates were measured at 0.5–2.5 cm depth in the grazed plots (115.0–319.6 µg CH_4_ oxidized per g dry soil and day, Fig. 3). The exclosed plots had highest CH_4_ oxidation rates at 3–8 cm depth (21.8–105.7 µg CH_4_ oxidized per g dry soil and day, Fig. 3). This shift in potential CH_4_ oxidation rates between the grazed and exclosed plots coincided with the shifts in O_2_ concentrations, water content and CH_4_ pore water concentrations. Overall higher CH_4_ oxidation rates were measured in grazed plots, exceeding 50 µg CH_4_ oxidized per g dry soil and day at most depths. In exclosed plots, CH_4_ oxidation rates higher than 50 µg per g dry soil and day were almost exclusively observed in the zones of maximal CH_4_ oxidation between 3 to 8 cm. The differences between grazed and exclosed plots, and different depths were true for both summer seasons (2015 and 2016) and the spring season. However, the potential CH_4_ oxidation rates in spring were overall lower than in summer for the grazed plots, while for the exclosed plots spring and summer CH_4_ oxidation rates were similar.

### Bacterial and MOB community structure

We then wanted to identify the main MOB taxa within the bacterial communities to specifically target the MOB responsible for the CH_4_ oxidation activity.

Larger amounts of DNA and RNA per gram dry soil were extracted from grazed plots compared to exclosed plots, suggesting a larger bacterial biomass in grazed soils (Fig. S4, Supporting Information). Sequencing of 16S rRNA gene libraries from these soils provided us with 10 816 sequences per library on average after quality filtering, the smallest library containing 2383 and the largest 19 608 sequences.

**Figure 4.**
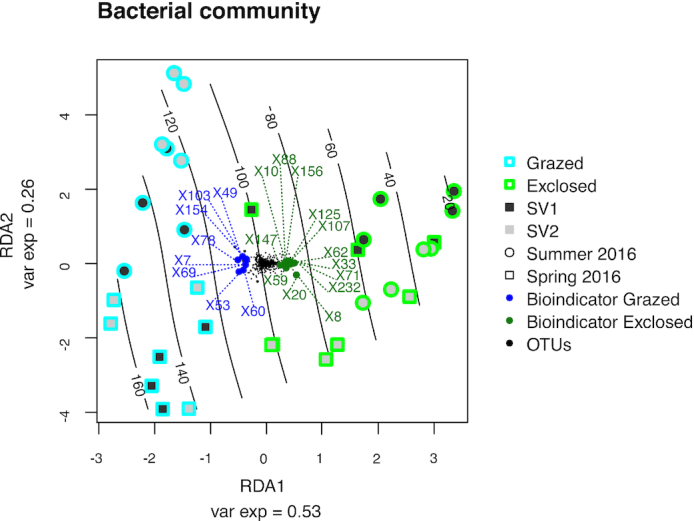
Treatment and season-dependent differences of bacterial communities at Solvatn peatland sites. The figure is based on redundancy analysis of the bacterial community (V3-V4 region of the 16S rRNA gene). Samples are labeled according to treatment: grazed (blue) and exclosed (green); sites: SV1 (dark grey) and SV2 (light grey); and sampling season: spring 2016 (square), summer 2016 (circle). Black lines indicate CH_4_ oxidation potentials (µg CH_4_ oxidized per g soil and day). Black dots show the distribution of non-bioindicator OTUs while green dots represent bioindicator OTUs for exclosed treatment and blue dots represent bioindicator OTUs for grazed treatment. Bioindicator identities are represented by the letter X followed by a number. Taxonomic information about the bioindicator OTUs is found in the V3-V4 heatmap (Fig. S6, Supporting Information).

The results of the RDA showed that the bacterial communities in grazed plots differed significantly from the communities in the exclosed plots (*P* < 0.001, Fig. 4). The RDA showed that the sampling date had an impact on the communities in the grazed plots (*P* = 0.004), which was less pronounced for exclosed plots (*P* = 0.124), similar as observed for the measured CH_4_ oxidation potentials.

The separation between grazed and exclosed plots correlated with *ex situ* CH_4_ oxidation rates and the communities in the grazed plots were associated with high CH_4_ oxidation rates while communities in the exclosed plots were associated with low rates (Fig. 4). However, CH_4_ oxidation rates did not have a significant impact on the community structure (*P* = 0.300 grazed plots, *P* = 0.673 exclosed plots, *P* = 0.613 both treatments). All p-values for the variables and variables’ interaction tested in the RDA analyses are listed in Table S4 (Supporting Information).

The 16S rRNA gene sequences assigned to the order Methylococcales were relatively more abundant (Kruskal-Wallis rank sum test *P* < 0.001) in grazed plots than in the exclosed plots, with a relative abundance of 7.0% in grazed plots compared to below 1% in exclosed plots (Fig. S5, Supporting Information). OTUs assigned to *Crenothrix* and *Methylobacter* had higher relative abundances than other genera within Methylococcales, representing 22 out of 25 OTUs of that order and from 56% to 100% of the sequences (Fig. S7, Supporting Information). Both genera were relatively more abundant in grazed plots (Kruskal–Wallis rank sum test: *P* < 0.05 for *Crenothrix* and for *Methylobacter P* < 0.01). From the 8 bioindicator OTUs for the grazed plots, one *Methylobacter* OTU (X49) was identified as bioindicator (Fig. 4, Fig. S6, Supporting Information). Among 13 OTUs identified as bioindicators for the exclosed plots, none of them belonged to the MOB.

To obtain further insights into the active MOB community, we sequenced *pmoA* transcript cDNA libraries from the summer 2015 microcosms and the spring and summer 2016 *in situ* soil samples using the two different *pmoA* primer pairs (Table S1, Supporting Information). The RDA analyses gave similar overall trends for both datasets. Therefore, the results from the mb661R *pmoA* dataset are used as the primary data basis for analyses, while the A682R *pmoA* dataset is used as a reference point to discuss uncertainties arising due to primer pair selection. Sequencing of *pmoA* gene libraries using the mb661R primer provided us with 29 149 sequences per library on average after quality filtering, the smallest library containing 9007 and the largest 58 612 sequences, whereas for the A682R primer an average of 22 340 sequences per library was provided, the smallest library containing 2213 and the largest library containing 53 950 sequences.

**Figure 5.**
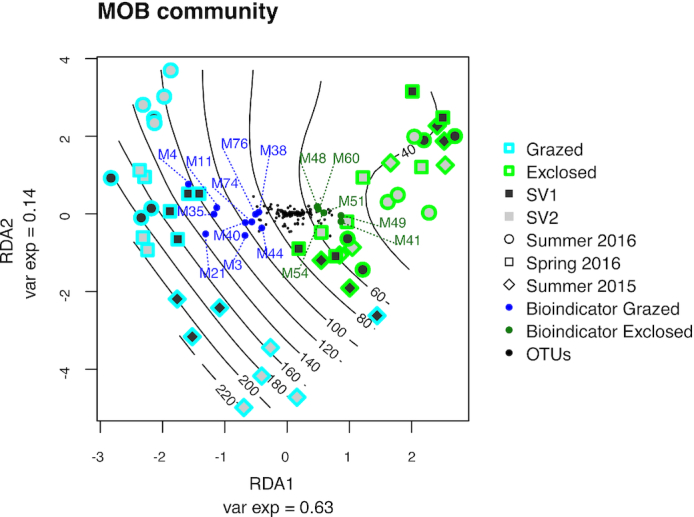
Treatment, site and season-dependent differences of MOB communities at Solvatn peatland sites. The figure is based on redundancy analysis of the MOB community (*pmoA* transcripts, primer pair A189F/mb661R). Samples are labeled according to treatment: grazed (blue) and exclosed (green); sites: SV1 (dark grey) and SV2 (light grey); and sampling season: summer 2015 (tilted square), spring 2016 (square), summer 2016 (circle). Black lines indicate CH_4_ oxidation potential (µg CH_4_ oxidized per g soil and day). Black dots show the distribution of non-bioindicator OTUs while green dots represent bioindicator OTUs for exclosed treatment and blue dots represent bioindicator OTUs for grazed treatment. Bioindicator identities are represented by the letter M followed by a number, marking them as OTUs from the mb661R *pmoA* dataset. Taxonomic information about the bioindicator OTUs is found in the heatmap in Fig. 6.

MOB community *pmoA* transcription in the grazed plots differed significantly from the exclosed plots (*P* < 0.001) (Fig. 5, Fig. S8, Table S4, Supporting Information), following the differences in CH_4_ oxidation rates. Sampling date also had a clear impact on the MOB *pmoA* expression in the grazed plots (*P* < 0.001), but not in the exclosed plots (*P* = 0.467).

**Figure 6.**
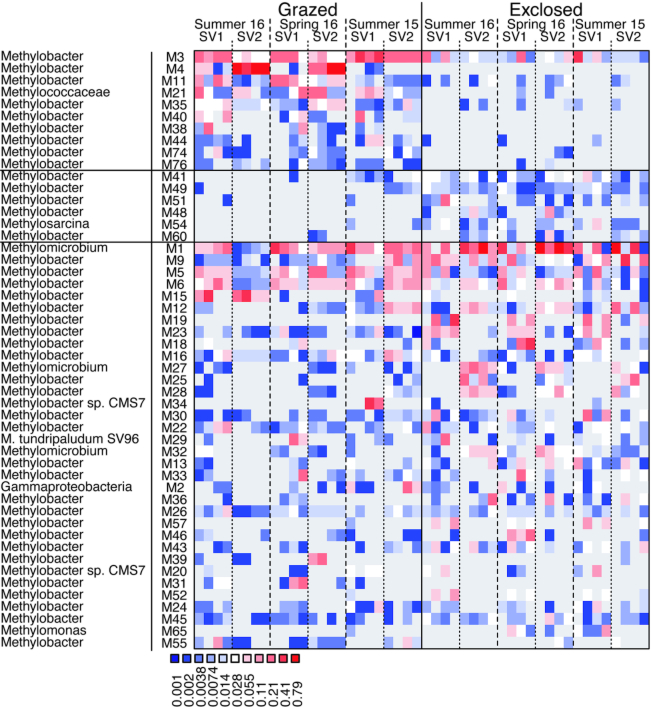
Relative abundances of MOB OTUs retrieved from *pmoA* transcripts *in situ* and *ex situ* (microcosm experiment). Bioindicator OTUs for the grazed treatment are shown in the uppermost section while the bioindicator OTUs for the exclosed treatment are shown in the middle section. In the lowest section we show the MOB OTUs with the highest relative abundance until representing 90% of the community. OTU names consist of the letter M plus a number, marking them as OTUs from the mb661R *pmoA* dataset. The color represents the relative abundance of a given OTU in a given sample.

The majority of *pmoA* transcripts belonged to the genus *Methylobacter* (Figs S9 and S10, Supporting Information). Furthermore, nearly all bioindicator OTUs (for grazed or exclosed plots) belonged to *Methylobacter* (Fig. 6). This indicates that a consortium of closely related species and strains within the same genus are primarily responsible for most of the CH_4_ oxidation but are also very responsive to ecosystem change.

OTUs from only two other genera, *Methylosarcina* and *Methylomicrobium*, were identified as bioindicators (OTU M54, Fig. 6 and OTUs A65 and A66, Fig. S11, Supporting Information). The relative abundance of *Methylosarcina* was low (< 1% in both *pmoA* datasets).

*Methylomicrobium* was the second most active genus based on the mb661R *pmoA* dataset (10.7% of the sequences in grazed plots, 27.6% in exclosed plots, Fig. S9, Supporting Information). OTU *Methylomicrobium*-M1 was the OTU with the highest overall abundance. However, the transcriptional activity of this and other *Methylomicrobium* OTUs were similar in all plots (Fig. 6) excluding those OTUs as bioindicator for grazed or exclosed plots.

MOB transcriptional profile based on the A682R *pmoA* dataset was slightly different from the mb661R *pmoA* dataset. The major discrepancy was the large amount of unidentified MOB annotated as MOB-like (Fig. S10, Supporting Information). OTU A6 had the highest relative abundance within the MOB-like group with no significant differences between the grazed and the exclosed plots, ranking fourth in relative abundance behind *Methylobacter* OTUs A1, A3 and A4.

**Figure 7.**
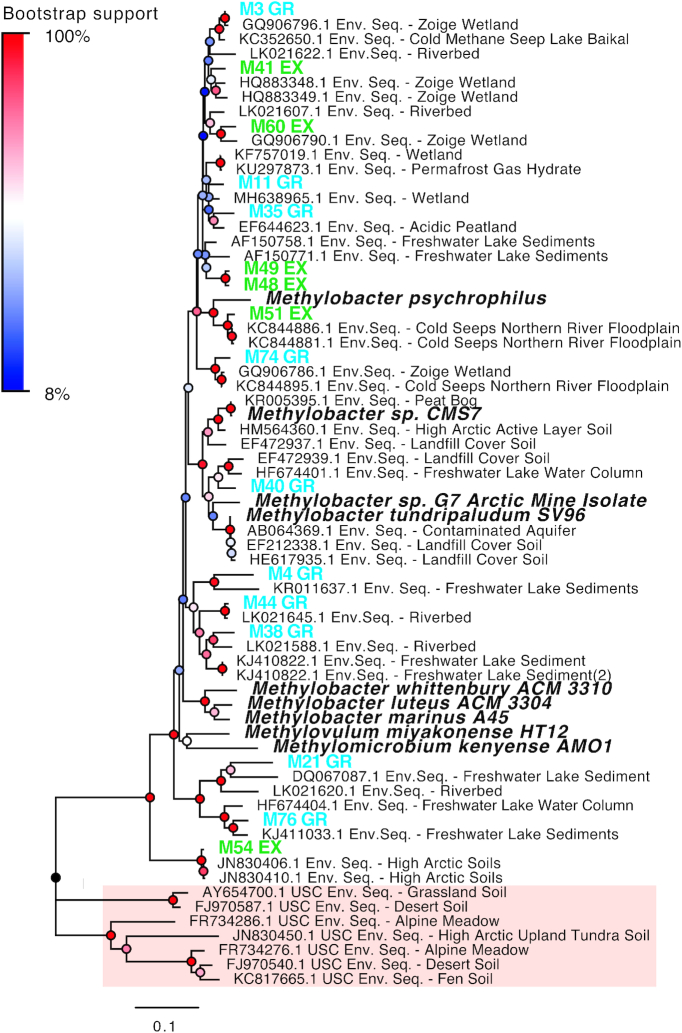
Neighbour-joining tree showing the phylogeny of the mb661R *pmoA* bioindicator OTUs (exclosed treatment in green, grazed treatment in blue), cultivated MOB strains (bold and italic) and related environmental sequences retrieved from Genbank (NCBI). Sequences belonging to the upland soil cluster gamma are used as outgroup to root the tree (shaded in red). Calculation was based on a 440-nucleotide alignment, using Jukes Cantor correction and 500 bootstraps. Bootstrap support is shown as node color ranging from blue (8%, lowest) to red (100%, highest). The length of the branches is based on the scale of 0.1 changes per nucleotide.

Phylogenetic analysis of the *pmoA* sequences of the bioindicator OTUs showed that most of them cluster within *Methylobacter*, in most cases closer to uncultivated environmental sequences than cultivated strains (Fig. 7). Interestingly, the bioindicators were all members of distinct clusters showing that these are phylogenetically different MOB strains.

## DISCUSSION

### Different ecosystem states change the potential for CH_4_ oxidation

In our study, we investigated how the presence or absence of herbivory affected the potential for CH_4_ oxidation and the methanotroph community in a high-Arctic peatland. Herbivore exclusion had promoted a higher proportion of vascular plants (Fig. 1) and less dense soil structure, reflected in higher O_2_ concentrations, lower water content and higher soil temperatures (Fig. 2, Table S3, Figs S1 and S2, Supporting Information). The higher temperatures may be explained by better insulation being provided by the thicker vascular plant cover (Sjögersten, Van Der Wal and Woodin 2008; Sjögersten *et al*. 2011; Falk *et al*. 2015). In addition, visual observations of living roots in the exclosed plots but not in the grazed plots confirmed previous observations of higher root and vascular plant biomass in the exclosed plots (Sjögersten *et al*. 2011). Lower *in situ* CH_4_ concentrations in the exclosed plots (Fig. S3, Supporting Information) contrast the higher temperatures measured, as one would expect increased microbial activity at higher temperatures. However, the higher O_2_ concentrations at 0–5 cm depth in the exclosed plots would promote CH_4_ oxidation and inhibit CH_4_ production, in line with our observations.

We did not observe any seasonally dependent differences in water content in grazed plots. Exclosed plots had overall lower water contents than the grazed plots and also slightly higher water contents in spring compared to summer, possibly due to recent snowmelt (Fig. 2).

The higher water content, higher *in situ* CH_4_ concentrations and lower O_2_ concentrations measured in the grazed plots were indicative of higher rates of CH_4_ production in these soils (Table S3, Fig. 2, Figs S2 and S3, Supporting Information). However, due to the higher SOM content available for microbial degradation in exclosed plots it cannot be excluded that the amount of CH_4_ produced in exclosed plots occasionally can surpass the CH_4_ produced in grazed plots.

The potential CH_4_ oxidation rates were highest in the grazed plots (Fig. 3) Furthermore, in these plots the zones of CH_4_ oxidation were closer to the peat surface in grazed plots. In contrast to this, the lower *in situ* CH_4_ concentrations and higher O_2_ concentrations in the exclosed plots corresponded to a vertical shift of the maximum CH_4_ oxidation zone from directly below the surface to between three and eight centimeters depth (Fig. 3). This matches the higher potential CH_4_ oxidation rates measured in grazed areas of alpine meadows (Abell *et al*. 2009) and smaller CH_4_ net-emissions measured in ungrazed areas of the Zackenberg valley (Greenland) and Yukon-Kuskokwim Delta (Western Alaska) (Falk *et al*. 2015; Kelsey *et al*. 2016). A possible explanation for the higher potential CH_4_ oxidation in the grazed sites is differences in nitrogen (N) availability. Ammonia (NH_4_) concentrations has been shown to correlate with higher CH_4_ oxidation rates and type I MOB abundances in rice paddy soils (Bodelier and Laanbroek 2004). Sjögersten *et al*. (2011) also showed higher N in grazed plots than in exclosed plots which can explain the higher CH_4_ oxidation observed in grazed plots in our study. Interestingly, it has been proposed that N fertilization favors a lower diversity (genus level) in rice paddy soils (Noll, Frenzel and Conrad 2008). As such, we would expect to observe higher methanotroph diversities in exclosed plots than grazed plots, but we did not.

### Different bacterial and methanotroph communities in grazed and exclosed soils

Related to the higher potential CH_4_ oxidation rates, we observed a considerably more transcriptionally active methanotroph community in grazed than exclosed plots (Fig. S6, Supporting Information). This difference was emphasized by the overall higher DNA and RNA content per gram dry soil in the zone of maximal CH_4_ oxidation in grazed peat soils compared to exclosed peat soils (Fig. S4, Supporting Information). Furthermore, the higher relative abundances of methanotrophs in the grazed plots corresponded with higher CH_4_ oxidation rates measured in the microcosm experiment *ex situ* (Fig. 3). Similarly, a link between CH_4_ oxidation rates and transcript abundances was previously demonstrated (Reim *et al*. 2012; Siljanen *et al*. 2012) but we did not apply RT-qPCR and cannot directly compare this.

*Methylobacter* made up the majority of the methanotroph communities at Solvatn. From the genomes of several *Methylobacter* species, we know that these microorganisms oxidize CH_4_ using the particulate methane monooxygenase. Our sequencing efforts targeting *pmoA* transcripts confirmed this, as the majority of *pmoA* transcripts were assigned to *Methylobacter* (Fig. 6 and Fig. S11, Supporting Information). Furthermore, we observed that a set of *Methylobacter* bioindicator OTUs were consistently more active in the maximal CH_4_ oxidation zone of the exclosed plots while other OTUs were more active in the grazed plots, indicating that the different CH_4_ and O_2_ concentrations favor different *Methylobacter* OTUs. Similiarly, in stratified lakes the MOB communities were structured according to CH_4_ and O_2_ concentrations, providing for a niche-adapted community responsible for CH_4_ oxidation dynamics (Mayr *et al*. 2020). An earlier microcosm study also supported the idea that closely related *Methylobacter* populations are adapted to different niches as they responded differently to O_2_ tension (Oshkin *et al*. 2015).

CH_4_ concentrations in Solvatn peat soil are high (Fig. S3, Supporting Information) while net net CH_4_ emissions are low (Høj, Olsen and Torsvik 2005; Sjögersten *et al*. 2011). It has previously been suggested that such niche partitioning increases the exploitation of resources (Finke and Snyder 2008; Mayr *et al*. 2020). Thus, niche partitioning of closely related *Methylobacter* OTUs may explain the efficiency of CH_4_ consumption in both grazed and exclosed peat soils at Solvatn.

Our study shows that phylogenetically distinct *Methylobacter* OTUs might find their ecological niches within micro niches of the same ecosystem as the most active fraction of the MOB community consisted of several closely related transcriptionally active *Methylobacter* populations (Figs. 6 and 7). Similar observations were reported from Lake Washington, Lake Pavin and the Canadian high Arctic (Costello and Lidstrom 1999; Martineau, Whyte and Greer 2010; Biderre-Petit *et al*. 2011), suggesting that our findings reflect a common occurrence. The bioindicator approach allows us to identify OTUs that respond to specific environmental changes in different ecosystems. By correlating past and future datasets this approach can help determining whether certain strains or species have the same roles and responses in other ecosystems or under other conditions.

It remains difficult to a draw a line between species and strains based on sequencing the *pmoA* genes or transcripts even though similarity cut-offs have been suggested for *pmoA* OTUs (Wen, Yang and Liebner 2016). A genome-based study revealed that a large variety of *Methylobacter* genomes which were earlier assigned as strains of *M. tundripaludum* SV96 are actually different species (Orata *et al*. 2018). Thus, some of the *pmoA* OTUs we have identified as bioindicators may be representative of different *Methylobacter* species, and so the OTU dynamics described here are in part reflecting the ecology of *Methylobacter* species.

## CONCLUSION

Herbivory in Svalbard leads to reduced vascular plant and root biomass in peatlands, resulting in increased soil water content, higher *in situ* pore water CH_4_ concentrations and reduced O_2_ concentrations. These changes correspond with a shallower and more potent zone of maximal CH_4_ oxidation in grazed peat compared to peat protected from grazing. Furthermore, the shallower CH_4_ oxidation zone in grazed peat has a relatively more abundant and different MOB community than the exclosed peat, the major difference being the dominance of different *Methylobacter* OTUs. Nevertheless, *Methylobacter* comprise the major CH_4_ filter in both peat soils, actively reducing the amount of CH_4_ emitted to the atmosphere. This study emphasizes how herbivory leads to altered soil conditions that selects for different active MOB communities able to respond to increased CH_4_ concentrations.

## Supplementary Material

fiaa140_Supplemental_File

## References

[bib1] Abell GCJ , Stralis-Pavese N, Sessitsch A et al. Grazing affects methanotroph activity and diversity in an alpine meadow soil. Environ Microbiol Rep. 2009;1:457–65. 23765900 10.1111/j.1758-2229.2009.00078.x

[bib2] Amaral JA , Knowles R. Growth of methanotrophs in methane and oxygen counter gradients. FEMS Microbiol Lett. 1995;126:215–20.

[bib3] Anderson MJ , Ellingsen KE, McArdle BH. Multivariate dispersion as a measure of beta diversity. Ecol Lett. 2006;9:683–93. 16706913 10.1111/j.1461-0248.2006.00926.x

[bib4] Bardgett RD , Manning P, Morriën E et al. Hierarchical responses of plant-soil interactions to climate change: Consequences for the global carbon cycle. J Ecol. 2013;101:334–43.

[bib5] Beck DAC , Kalyuzhnaya MG, Malfatti S et al. A metagenomic insight into freshwater methane-utilizing communities and evidence for cooperation between the Methylococcaceae and the Methylophilaceae. Peer J. 2013;2013:1–23. 10.7717/peerj.23 PMC3628875 23638358

[bib6] Biderre-Petit C , Jézéquel D, Dugat-Bony E et al. Identification of microbial communities involved in the methane cycle of a freshwater meromictic lake. FEMS Microbiol Ecol. 2011;77:533–45. 21595728 10.1111/j.1574-6941.2011.01134.x

[bib7] Blees J , Niemann H, Wenk CB et al. Micro-aerobic bacterial methane oxidation in the chemocline and anoxic water column of deep south-alpine Lake Lugano (Switzerland). Limnol Oceanogr. 2014;59:311–24.

[bib8] Bodelier PLE , Laanbroek HJ. Nitrogen as a regulatory factor of methane oxidation in soils and sediments. FEMS Microbiol Ecol. 2004;47:265–77. 19712315 10.1016/S0168-6496(03)00304-0

[bib10] Bornemann M , Bussmann I, Tichy L et al. Methane release from sediment seeps to the atmosphere is counteracted by highly active Methylococcaceae in the water column of deep oligotrophic Lake Constance. FEMS Microbiol Ecol. 2016;92:1–11. 10.1093/femsec/fiw123 27267930

[bib9] Börjesson G , Sundh I, Svensson B. Microbial oxidation of CH4 at different temperatures in landfill cover soils. FEMS Microbiol Ecol. 2004;48:305–12. 19712300 10.1016/j.femsec.2004.02.006

[bib11] Costello AM , Lidstrom ME. Molecular characterization of functional and phylogenetic genes from natural populations of methanotrophs in lake sediments. Appl Environ Microbiol. 1999;65:5066–74. 10543824 10.1128/aem.65.11.5066-5074.1999 PMC91682

[bib12] Edgar RC . MUSCLE: Multiple sequence alignment with high accuracy and high throughput. Nucleic Acids Res. 2004;32:1792–7. 15034147 10.1093/nar/gkh340 PMC390337

[bib13] Falk JM , Schmidt NM, Christensen TR et al. Large herbivore grazing affects the vegetation structure and greenhouse gas balance in a high Arctic mire. Environ Res Lett. 2015;10:045001.

[bib14] Finke DL , Snyder WE. Niche partitioning increases resource exploitation by diverse communities. Science (80-). 2008;321:1488–90. 10.1126/science.1160854 18787167

[bib15] Graef C , Hestnes AG, Svenning MM et al. The active methanotrophic community in a wetland from the High Arctic. Environ Microbiol Rep. 2011;3:466–72. 23761309 10.1111/j.1758-2229.2010.00237.x

[bib16] Hanson RS , Hanson TE. Methanotrophic bacteria. Microbiol Rev. 1996;60:439–71. 8801441 10.1128/mr.60.2.439-471.1996 PMC239451

[bib17] He R , Wooller MJ, Pohlman JW et al. Identification of functionally active aerobic methanotrophs in sediments from an Arctic lake using stable isotope probing. Environ Microbiol. 2012;14:1403–19. 22429394 10.1111/j.1462-2920.2012.02725.x

[bib18] Hessen DO , Tombre IM, van Geest G et al. Global change and ecosystem connectivity: how geese link fields of central Europe to eutrophication of Arctic freshwaters. Ambio. 2017;46:40–7. 27352361 10.1007/s13280-016-0802-9 PMC5226897

[bib19] Ho A , Kerckhof F-M, Lüke C et al. Conceptualizing functional traits and ecological characteristics of methane-oxidizing bacteria as life strategies. Environ Microbiol Rep. 2013;5:335–45. 23754714 10.1111/j.1758-2229.2012.00370.x

[bib21] Holmes AJ , Costello A, Lidstrom ME et al. Evidence that particulate methane monooxygenase and ammonia monooxygenase may be evolutionarily related. FEMS Microbiol Lett. 1995;132:203–8. 7590173 10.1016/0378-1097(95)00311-r

[bib20] Høj L , Olsen RA, Torsvik VL. Archaeal communities in high-Arctic wetlands at Spitsbergen, Norway (78°N) as characterized by 16S rRNA gene fingerprinting. FEMS Microbiol Ecol. 2005;53:89–101. 16329932 10.1016/j.femsec.2005.01.004

[bib22] Kelsey KC , Leffler AJ, Beard KH et al. Interactions among vegetation, climate, and herbivory control greenhouse gas fluxes in a subarctic coastal wetland. J Geophys Res Biogeosciences. 2016;121:2960–75.

[bib23] Klindworth A , Pruesse E, Schweer T et al. Evaluation of general 16S ribosomal RNA gene PCR primers for classical and next-generation sequencing-based diversity studies. Nucleic Acids Res. 2013;41:1–11. 22933715 10.1093/nar/gks808 PMC3592464

[bib24] Knief C . Diversity and habitat preferences of cultivated and uncultivated aerobic methanotrophic bacteria evaluated based on pmoA as molecular marker. Front Microbiol. 2015;6:1–38. 26696968 10.3389/fmicb.2015.01346 PMC4678205

[bib25] Kruse J , Lennartz B, Leinweber P. A modified method for measuring saturated hydraulic conductivity and anisotropy of fen peat samples. Wetlands. 2008;28:527–31.

[bib26] Kumar S , Stecher G, Tamura K. MEGA7: Molecular evolutionary genetics analysis version 7.0 for bigger datasets. Mol Biol Evol. 2016;33:1870–4. 27004904 10.1093/molbev/msw054 PMC8210823

[bib27] Lane DJ . 16S/23S rRNA sequencing. Nucleic Acid Techniques in Bacterial Systematics, New York: John Wiley & Sons, 1991, 115–75.

[bib28] Liebner S , Schwarzenbach SP, Zeyer J. Methane emissions from an alpine fen in central Switzerland. Biogeochemistry. 2012;109:287–99.

[bib29] Magoč T , Salzberg SL. FLASH: Fast length adjustment of short reads to improve genome assemblies. Bioinformatics. 2011;27:2957–63. 21903629 10.1093/bioinformatics/btr507 PMC3198573

[bib30] Mahé F , Rognes T, Quince C et al. Swarm: robust and fast clustering method for amplicon-based studies. Peer J. 2014;2, DOI:10.7717/peerj.593. PMC4178461 25276506

[bib31] Martineau C , Whyte LG, Greer CW. Stable isotope probing analysis of the diversity and activity of Methanotrophic bacteria in soils from the Canadian high Arctic. Appl Environ Microbiol. 2010;76:5773–84. 20622133 10.1128/AEM.03094-09 PMC2935073

[bib32] Mayr MJ , Zimmermann M, Guggenheim C et al. Niche partitioning of methane-oxidizing bacteria in the oxygen-methane counter gradient of stratified lakes. ISME J. 2020;14:274–87. 31624343 10.1038/s41396-019-0515-8 PMC6908591

[bib33] McDonald IR , Bodrossy L, Chen Y et al. Molecular ecology techniques for the study of aerobic methanotrophs. Appl Environ Microbiol. 2008;74:1305–15. 18165358 10.1128/AEM.02233-07 PMC2258629

[bib34] Niemann H , Steinle L, Blees J et al. Toxic effects of lab-grade butyl rubber stoppers on aerobic methane oxidation. Limnol Oceanogr Methods. 2015;13:40–52.

[bib35] Noll M , Frenzel P, Conrad R. Selective stimulation of type I methanotrophs in a rice paddy soil by urea fertilization revealed by RNA-based stable isotope probing. FEMS Microbiol Ecol. 2008;65:125–32. 18544098 10.1111/j.1574-6941.2008.00497.x

[bib36] Oksanen J , Blanchet FG, Friendly M et al. vegan: Community Ecology Package. R. 2018, ISBN: 0-387-95457-0.

[bib37] Orata FD , Meier-Kolthoff JP, Sauvageau D et al. Phylogenomic analysis of the gammaproteobacterial Methanotrophs (order Methylococcales) calls for the reclassification of members at the genus and species evels. Front Microbiol. 2018;9:1–17. 30631317 10.3389/fmicb.2018.03162 PMC6315193

[bib38] Oshkin IY , Beck DAC, Lamb AE et al. Methane-fed microbial microcosms show differential community dynamics and pinpoint taxa involved in communal response. ISME J. 2015;9:1119–29. 25333464 10.1038/ismej.2014.203 PMC4409156

[bib39] Oshkin IY , Wegner C-E, Lüke C et al. Gammaproteobacterial methanotrophs dominate cold methane seeps in floodplains of west siberian rivers. Appl Environ Microbiol. 2014;80:5944–54. 25063667 10.1128/AEM.01539-14 PMC4178702

[bib40] Oswald K , Milucka J, Brand A et al. Light-dependent aerobic methane oxidation reduces methane emissions from seasonally stratified lakes. PLoS One. 2015;10:e0132574. 26193458 10.1371/journal.pone.0132574 PMC4508055

[bib41] Parish F , Sirin A, Charman D et al. Assessment on Peatlands, Biodiversity and Climate Change: Main Report. Global Environment Centre, Kuala Lumpur and Wetlands International, Wageningen, 2008, 179.

[bib42] Pearson WR . Flexible sequence similarity searching with the FASTA3 program package. In: Misener S, Krawetz SA, (eds.). Methods in Molecular Biology, Totowa, NJ: Humana Press, 2000, 185–219. 10.1385/1-59259-192-2:185 10547837

[bib43] Quast C , Pruesse E, Yilmaz P et al. The SILVA ribosomal RNA gene database project: Improved data processing and web-based tools. Nucleic Acids Res. 2013;41:590–6. 10.1093/nar/gks1219 PMC3531112 23193283

[bib45] Rambaut A . FigTree, v. 1.4.4. 2018.

[bib44] R Core Team. R Core Team (2018). R: A language and environment for statistical computing. R Foundation for statistical computing, Vienna, Austria. Available online at https://www.R-project.org/. 2018.

[bib46] Reay DS , Smith KA, Hewitt CN. Methane: importance, sources and sinks. In: Reay DS, Hewitt CN, Smith KA, Grace J, (eds.). Greenhouse Gas Sinks, Wallingford, Oxfordshire, UK: CABI, 2007, 143–51.

[bib47] Reim A , Lüke C, Krause S et al. One millimetre makes the difference: high-resolution analysis of methane-oxidizing bacteria and their specific activity at the oxic-anoxic interface in a flooded paddy soil. ISME J. 2012;6:2128–39. 22695859 10.1038/ismej.2012.57 PMC3475382

[bib48] Roberts D . Package ‘ labdsv.’. 2016.

[bib49] Rognes T , Flouri T, Nichols B et al. VSEARCH: a versatile open source tool for metagenomics. Peer J. 2016;4:e2584. 27781170 10.7717/peerj.2584 PMC5075697

[bib50] Semrau JD , Chistoserdov A, Lebron J et al. Particulate methane monooxygenase genes in methanotrophs. J Bacteriol. 1995;177:3071–9. 7768803 10.1128/jb.177.11.3071-3079.1995 PMC176995

[bib51] Semrau JD , DiSpirito AA, Yoon S. Methanotrophs and copper. FEMS Microbiol Rev. 2010;34:496–531. 20236329 10.1111/j.1574-6976.2010.00212.x

[bib52] Siljanen HMP , Saari A, Bodrossy L et al. Seasonal variation in the function and diversity of methanotrophs in the littoral wetland of a boreal eutrophic lake. FEMS Microbiol Ecol. 2012;80:548–55. 22296339 10.1111/j.1574-6941.2012.01321.x

[bib53] Singleton CM , McCalley CK, Woodcroft BJ et al. Methanotrophy across a natural permafrost thaw environment. ISME J. 2018;12:2544–58. 29955139 10.1038/s41396-018-0065-5 PMC6155033

[bib54] Sjögersten S , van der Wal R, Loonen MJJE et al. Recovery of ecosystem carbon fluxes and storage from herbivory. Biogeochemistry. 2011;106:357–70. 26069352 10.1007/s10533-010-9516-4 PMC4459552

[bib55] Sjögersten S , Van Der Wal R, Woodin SJ. Habitat type determines herbivory controls over CO2 fluxes in a warmer Arctic. Ecology. 2008;89:2103–16. 18724721 10.1890/07-1601.1

[bib56] Smith GJ , Angle JC, Solden LM et al. Members of the genus Methylobacter are inferred to account for the majority of aerobic methane oxidation in oxic soils from a freshwater eetland. MBio. 2018;9:e00815–18. 30401770 10.1128/mBio.00815-18 PMC6222125

[bib57] Solheim B , Endal A, Vigstad H. Nitrogen fixation in Arctic vegetation and soils from Svalbard, Norway. Polar Biol. 1996;16:35–40.

[bib58] Stępniewska Z , Goraj W, Kuźniar A et al. Methane oxidation by endophytic bacteria inhabiting Sphagnum sp. and some vascular plants. Wetlands. 2018;38:411–22.

[bib59] Tsutsumi M , Iwata T, Kojima H et al. Spatiotemporal variations in an assemblage of closely related planktonic aerobic methanotrophs. Freshw Biol. 2011;56:342–51.

[bib60] Tveit A , Schwacke R, Svenning MM et al. Organic carbon transformations in high-Arctic peat soils: key functions and microorganisms. ISME J. 2013;7:299–311. 22955232 10.1038/ismej.2012.99 PMC3554415

[bib61] Tveit AT , Urich T, Frenzel P et al. Metabolic and trophic interactions modulate methane production by Arctic peat microbiota in response to warming. Proc Natl Acad Sci. 2015;112:E2507–16. 25918393 10.1073/pnas.1420797112 PMC4434766

[bib62] Urich T , Lanzén A, Qi J et al. Simultaneous assessment of soil microbial community structure and function through analysis of the meta-transcriptome. PLoS One. 2008;3:e2529. 18575584 10.1371/journal.pone.0002527 PMC2424134

[bib63] Wartiainen I , Hestnes AG, McDonald IR et al. Methylobacter tundripaludum sp. nov., a methane-oxidizing bacterium from Arctic wetland soil on the Svalbard islands, Norway (78° N). Int J Syst Evol Microbiol. 2006;56:109–13. 16403874 10.1099/ijs.0.63728-0

[bib64] Wartiainen I , Hestnes AG, Svenning MM. Methanotrophic diversity in high arctic wetlands on the islands of Svalbard (Norway)–denaturing gradient gel electrophoresis analysis of soil DNA and enrichment cultures. Can J Microbiol. 2003;49:602–12. 14663494 10.1139/w03-080

[bib65] Wen X , Yang S, Liebner S. Evaluation and update of cutoff values for methanotrophic pmoA gene sequences. Arch Microbiol. 2016;198:629–36. 27098810 10.1007/s00203-016-1222-8

[bib66] Yun J , Ma A, Li Y et al. Diversity of methanotrophs in Zoige wetland soils under both anaerobic and aerobic conditions. J Environ Sci. 2010;22:1232–8. 10.1016/s1001-0742(09)60243-6 21179963

[bib67] Zhang L , Adams JM, Dumont MG et al. Distinct methanotrophic communities exist in habitats with different soil water contents. Soil Biol Biochem. 2019;132:143–52.

