# Supplementary material for: Methanotroph populations and CH4 oxidation potentials in high-Arctic peat are altered by herbivory induced vegetation change
Source: FEMS Microbiol Ecol. 2020 Jul 8;96(10):fiaa140. doi: 10.1093/femsec/fiaa140 (PMC8202349; doi:10.1093/femsec/fiaa140)
Supplement: fiaa140_Supplemental_File [file fiaa140_supplemental_file.docx]

**Supplementary Table 1: Sampling overview**

|  | **Summer 2015** | **Spring 2016** | **Summer 2016** |
| --- | --- | --- | --- |
| *In situ* soil parameters (temperature, O_2_ and CH_4_ concentration) | X | X | X |
| *Ex situ* soil parameters (soil water content, soil organic matter content) | X | X | X |
| CH_4_ oxidation measurements *ex situ* (microcosms) | X | X | X |
| 16S rRNA gene analysis of *in situ* bacterial community |  | X | X |
| *pmoA* transcript analysis of *in situ* MOB community |  | X | X |
| *pmoA* transcript analysis of *ex situ* MOB community (microcosms) | X |  |  |

**Supplementary Table 2: Pipeline overview for V3-V4 and *pmoA* (with both primer sets) amplicon sequence analysis**. Steps 1-5 describe the processing of raw sequences until Swarm clustering and are therefore not applicable (n/a) for OTU counts, which is described in step 7 and 8. Sequences from the V3-V4 dataset were not checked for frameshift mutations and step 5 is therefore not applicable (n/a).

|  |  | V3V4 | | mb661 | | A682 | |
| --- | --- | --- | --- | --- | --- | --- | --- |
|  |  | Sequences | OTUs | Sequences | OTUs | Sequences | OTUs |
| 1 | raw sequences | 1707324 | (n/a) | 2635949 | (n/a) | 2869266 | (n/a) |
| 2 | good quality | 706371 | (n/a) | 1606840 | (n/a) | 1189725 | (n/a) |
| 3 | with good primers | 647378 | (n/a) | 1567342 | (n/a) | 1162401 | (n/a) |
| 4 | not chimera | 352308 | (n/a) | 1496771 | (n/a) | 1151186 | (n/a) |
| 5 | respecting frameshift | (n/a) | (n/a) | 1399165 | (n/a) | 1073560 | (n/a) |
| 6 | clustering | 352197 | 53468 | 1399164 | 15909 | 1073533 | 13942 |
| 7 | good sequence length and taxonomy | 346132 | 52629 | 1399144 | 15897 | 1072313 | 13727 |
| 8 | high occurrence | 155893 | 1310 | 1366774 | 220 | 1044851 | 223 |

**Supplementary Fig. 1A and 1B: Soil temperatures measured along vertical soil gradients comparing grazed treatment (two top figures) and exclosed treatment (two bottom figures) in spring (left hand side) and summer (right hand side)**. Whiskers indicate min and max temperature measured; boxes consist of 25% quartile, median and 75% quartile.

**Supplementary Fig. 2: O_2_ profiles along vertical soil gradients (y-axis) comparing grazed treatment (left) and exclosed treatment (right**). Measurements were taken in 5 cm intervals in summer 2015, spring 2016 and summer 2016 at both sites, SV1 and SV2. Whiskers indicate min and max O_2_ concentration measured; boxes consist of 25% quartile, median and 75% quartile.

**Supplementary Fig. 3: CH_4_ pore water concentration measurements [mg/L] along vertical soil gradients (y-axis) comparing grazed treatment (left) and exclosed treatment (right)**. The data used in this figure are from summer 2015, spring 2016 and summer 2016 and were taken at both sites, SV1 and SV2. Whiskers indicate max and min CH_4_ concentrations; Boxes consist of 25% quartile, median and 75% quartile.

**Supplementary Fig. 4: RNA and DNA extracted per gram dry soil for grazed (white) and exclosed (grey) treatments as indicator for community size**. Whiskers indicate min. and max. values; boxes consist of 25% quartile, median and 75% quartile.

**Supplementary Table 3: Soil parameter ranges for grazed treatment (left) and exclosed treatment (right). Measurements at a depth of 0 cm are taken at the soil/vegetation interface.**

|  | Grazed | | | | | | Exclosed | | | | | |
| --- | --- | --- | --- | --- | --- | --- | --- | --- | --- | --- | --- | --- |
|  | **Spring** | | | **Summer** | | | **Spring** | | | **Summer** | | |
| Depth  [cm] | Temp.  [°C] | O_2_  [mg/L] | CH_4_  [mg/L] | Temp.  [°C] | O_2_  [mg/L] | CH_4_  [mg/L] | Temp.  [°C] | O_2_  [mg/L] | CH_4_  [mg/L] | Temp.  [°C] | O_2_  [mg/L] | CH_4_  [mg/L] |
|  |  |  |  |  |  |  |  |  |  |  |  |  |
| 0 | 5.7-11.0 | 10.9-11.5 | - | 8.3-16.1 | 9.8-11.3 | - | 5.2-12.0 | 10.4-11.5 | - | 7.0-16.0 | 10.7-11.6 | - |
| 5 | 4.2-7.8 | 1.7-4.5 | 1.0-2.0 | 5.4-10.9 | 7.1-9.5 | 0.2-3.8 | 1.4-5.7 | 9.6-12.1 | - | 4.4-9.8 | 11.0-11.4 | <0.1 |
| 10 | 3.0-5.1 | 1.5-2.3 | 3.3-8.1 | 4.5-8.4 | 0.2-2.4 | 1.7-34.3 | 0.4-3.9 | 4.0-12.3 | < 0.1-1.8 | 3.8-7.3 | 8.1-9.9 | < 0.1-0.1 |
| 15 | 1.7-3.5 | 0.6-2.2 | 4.6-7.5 | 3.4-6.4 | 1.0-1.9 | 2.1-10.4 | 1.0-2.5 | 7.4 | < 0.1-1.4 | 3.3-6.3 | 4.4 | <0.1-9.4 |
| 20 | 0.4-2.2 | 0.1-1.2 | 5.5-6.6 | 3.0-5.4 | 0.7-1.3 | 1.8-25.1 | 0.4-2.1 | 3.6 | - | 2.4-5.3 | 0.5 | 1.1-3.2 |
|  |  |  |  |  |  |  |  |  |  |  |  |  |

**Supplementary Table 4: p-value overview from RDA for each dataset (*pmoA*: mb661, A682; V3-V4).** Community column is split in total (all samples), grazed (samples from grazed treatment only) and exclosed (samples from exclosed treatment only). Sampling date shows the effect significance of the sampling period, Grazing shows the effect significance of the treatment and CH_4_ Oxidation Rate shows the relation significance between the potential CH_4_ oxidation measured in the microcosms and the communities. The last column shows the interaction between Sampling Date and Grazing, Site and Grazing. Interactions between the effect of Sampling Date and Grazing are not applicable (n/a) for the grazed and exclosed sub-datasets. Numbers lower then 0.05 show high interaction whereas numbers higher than 0.05 indicate low interaction.

| **Sequence Dataset** | **Community** | **Sampling Date** | **Grazing** | **CH_4_ Ox. Rate** | **Interaction - Sampling Date:Grazing** |
| --- | --- | --- | --- | --- | --- |
| V3V4 | Total | 0.007 | <0.001 | 0.613 | 0.130 |
|  | Grazed | 0.004 | (n/a) | 0.300 | (n/a) |
|  | Exclosed | 0.124 | (n/a) | 0.673 | (n/a) |
| mb661 | Total | 0.033 | <0.001 | 0.651 | 0.099 |
|  | Grazed | <0.001 | (n/a) | 0.277 | (n/a) |
|  | Exclosed | 0.467 | (n/a) | 0.149 | (n/a) |
| A682 | Total | 0.019 | <0.001 | 0.614 | 0.051 |
|  | Grazed | <0.001 | (n/a) | 0.230 | (n/a) |
|  | Exclosed | 0.453 | (n/a) | 0.270 | (n/a) |

**Supplementary Fig. 5: Distribution of bacterial taxa in grazed and exclosed treatments**. Only taxa with a relative abundance > 1% are represented. Relative abundances are listed in the outermost ring of the chart and are given in %. The different rings represent different taxonomic levels, starting with phyla in the innermost ring followed by class and order moving outward.

**Supplementary Fig. 6: Relative abundances of bacterial OTUs retrieved from 16S rRNA genes *in situ***. Bioindicator OTUs for the grazed treatment are shown in the uppermost section while the bioindicator OTUs for the exclosed treatment are shown in the middle section. In the lowest section we show the OTUs with the highest relative abundances until representing 50% of the community. OTU names consist of the letter X plus a number, marking them as OTUs from the V3-V4 dataset. The color represents the relative abundance of a given OTU in a given sample.

**Supplementary Fig. 7: Heatmap showing the increase in OTU abundance *in situ* assigned to the order Methylococcales in grazed treatment, which is visible in Supplementary Figs 5 and 6**. OTU names consist of the letter X plus a number, marking them as OTUs from the V3V4 dataset. The color represents the relative abundance of a given OTU in a given sample.

**Supplementary Fig.8: Treatment and season-dependent differences in the MOB communities at Solvatn peatland sites.** The figure is based on redundancy analysis of the MOB community (*pmoA* transcripts, primer pair A189F/A682R). Samples are labeled according to treatment: grazed (blue) and exclosed (green); sites: SV1 (dark grey) and SV2 (light grey); and sampling season: summer 2015 (tilted square), spring 2016 (square), summer 2016 (circle). Black lines indicate CH_4_ oxidation potential (µg CH_4_ oxidized per g soil and day). Black dots show the distribution of non-bioindicator OTUs, while green dots represent bioindicator OTUs for exclosed treatment and blue dots represent bioindicator OTUs for grazed treatment. Bioindicator identities are represented by the letter A followed by a number, marking them as OTUs from the A682R *pmoA* dataset. Taxonomic information can be obtained from the heatmap in Supplementary Fig. 11.

**Supplementary Fig. 9: Distribution of MOB taxa (A189F/mb661R primer) in grazed and exclosed treatments**. Only taxa with a relative abundance >1% are represented. Relative abundances are listed in the outermost ring of the chart and are given in %. The different rings represent different taxonomic levels, with genus in the innermost ring followed by species in the outermost ring.

**Supplementary Fig. 10: Distribution of MOB taxa (A189F/A682R primer) in grazed and exclosed treatments**. Only taxa with a relative abundance >1% were included. Relative abundances are listed in the outermost ring of the chart and are given in %. The different rings represent different taxonomic levels, with genus in the innermost ring followed by species in the outermost ring.

**Supplementary Fig. 11: Relative abundances of MOB OTUs retrieved from *pmoA* transcripts *in situ* and *ex situ* (microcosm experiment)**. Bioindicator OTUs for the grazed treatment are shown in the uppermost section while the bioindicator OTUs for the exclosed treatment are shown in the middle section. In the lowest section we show the MOB OTUs with the highest relative abundance until representing 90% of the community. OTU names consist of the letter A plus a number, indicating them as OTUs from the A682R *pmoA* dataset. The color represents the relative abundance of a given OTU in a given sample
